# Complete Genome Sequence of *Streptomyces* Phage Shaeky

**DOI:** 10.1128/MRA.01338-20

**Published:** 2021-01-07

**Authors:** Shae Shodrock, Tyler Higbee, James Clark, Isla Hernandez, Mei Liu, Ben Burrowes

**Affiliations:** a Department of Biochemistry and Biophysics, Texas A&M University, College Station, Texas, USA; b Center for Phage Technology, Texas A&M University, College Station, Texas, USA; Queens College

## Abstract

Here, we present the genome of siphophage Shaeky, infecting the Gram-positive bacterium *Streptomyces* sp. strain Mg1. Shaeky has very low sequence identity to other phages, with phage phiC31 being the most closely related in the NCBI database. The Shaeky genome is 45,617 bp with 77 protein-coding genes and 16 tRNAs.

## ANNOUNCEMENT

Members of *Streptomyces*, the largest genus in the phylum *Actinobacteria*, are characterized as filamentous Gram-positive bacteria. Many of the species are responsible for the decomposition of organic materials in soil (1). Certain species can colonize the respiratory tract and lead to a pulmonary disease known as Cushing syndrome (2).

Bacteriophage Shaeky was isolated by plaque purification, as described previously (3), in February 2019 from a soil sample taken from San Jordan, Utah, using the host strain *Streptomyces* sp. strain Mg1 grown at 30°C on nutrient broth with 10 mM MgCl_2_, 8 mM Ca(NO_3_)_2_, and 0.5% glucose. DNA was purified as described previously (4) using a Wizard DNA clean-up kit (Promega) and was prepared as Illumina TruSeq libraries with 300-bp inserts using a Nextera Flex kit. Sequencing was performed on an Illumina MiSeq system with paired-end 350-bp reads using 500-cycle v2 chemistry. The sequencing reads were quality controlled using FastQC (www.bioinformatics.babraham.ac.uk/projects/fastqc) and then manually trimmed with FastX v0.0.14 (http://hannonlab.cshl.edu/fastx_toolkit/download.html). The final genome was closed using PCR and Sanger sequencing with forward and reverse primers CGTTGAAGCCGCGATTG and TTCTGCCCGAGTTCGTG, respectively. The closed genome was found to have the size of 45,672 bp, with 384.6× contig sequencing coverage, using SPAdes v3.5.0; there were 415,598 total sequencing reads (5). Structural annotations were performed using GLIMMER v3 (6) and MetaGeneAnnotator v1.0 (7). tRNAs were discovered using ARAGORN v2.36 (8). Functionalities of genes were predicted using InterProScan v5.33 (9), TMHMM v2.0 (10), HHPred (11), and BLAST v2.9.0 (12) with the NCBI nonredundant, Swiss-Prot, and TrEMBL databases (13), with default settings (accessed 21 April 2020). The annotation tools described are available on the Galaxy server (https://cpt.tamu.edu/galaxy-pub) (14–16).

Genomic analysis predicts phage Shaeky to be a siphophage with a 45,617-bp double-stranded DNA (dsDNA) genome, 77 protein-coding genes, and 16 tRNAs. There were no predicted genome termini.

Of the 77 hypothetical protein-coding genes, 30 had predicted functions while 47 were novel proteins. The 16 tRNAs are located in one sequential stretch that spans ∼1.5 kb of the genome. No introns were found. BLASTn analysis shows that Phage Shaeky has only 16.9% nucleotide identity to phage phiC31 (GenBank accession number AJ006589.3), a *Streptomyces* temperate phage to which Shaeky is most closely related, and shares 25 unique proteins with *Streptomyces* phage Attoomi (GenBank accession number NC_047905.1). Therefore, Shaeky appears to be a novel siphovirus. All genes in the lysis cassette were identified except for spannins, which are expected for siphophages with Gram-positive hosts. Three predicted HNH endonuclease genes were found, one being downstream from the span of tRNAs and the other two located toward the beginning of the genome. There is an ∼7-kb span in the genome in which all protein-coding genes appear to be novel.

### Data availability.

The genome of Shaeky was deposited in GenBank with accession number MT701595.1. The associated BioProject, SRA, and BioSample accession numbers are PRJNA222858, SRR11558340, and SAMN14609632, respectively.
